# Proficiency in Motivational Interviewing among Nurses in Child Health Services Following Workshop and Supervision with Systematic Feedback

**DOI:** 10.1371/journal.pone.0163624

**Published:** 2016-09-29

**Authors:** Johanna Enö Persson, Benjamin Bohman, Lars Forsberg, Maria Beckman, Per Tynelius, Finn Rasmussen, Ata Ghaderi

**Affiliations:** 1 Department of Clinical Neuroscience, Karolinska Institutet, Stockholm, Sweden; 2 Centre for Psychiatric Research, Stockholm Health Care Services, Stockholm, Sweden; 3 MIC Lab AB, Stockholm, Sweden; 4 Child and Adolescent Public Health Epidemiology, Department of Public Health Sciences, Karolinska Institutet, Stockholm, Sweden; 5 Centre for Epidemiology and Community Medicine, Stockholm County Council, Health Care Services, Stockholm, Sweden; King Saud University, SAUDI ARABIA

## Abstract

**Background:**

Research on training in motivational interviewing (MI) has shown eroding skills after workshops not followed by additional training input (supervision/coaching). There is a need for more research evaluating different types and lengths of post-workshop training with follow-up periods extending six months. This study is an extension of a previous evaluation of the level of proficiency in MI after workshop and four sessions of supervision among nurses in Swedish child health services.

**Aims:**

To explore the level of MI proficiency among nurses participating in an intervention to prevent childhood obesity (*n* = 33), after receiving five additional sessions of supervision including feedback on observed practice, as well as level of proficiency at follow-up.

**Methods:**

Level of proficiency was measured 4 and 12 months after completed supervision using recorded practice samples coded according to the Motivational Interviewing Treatment Integrity (MITI) Code. Potential predictors of outcome were investigated.

**Results:**

Proficiency remained on the same levels after nine sessions of supervision as after four sessions, and was generally low. The percentage of nurses reaching the proficiency level ranged from 18.2 to 54.5% across indicators. MI-spirit had increased significantly at follow-up, and the rest of the indicators remained on the same levels. No predictors of outcome were found.

**Conclusions:**

Comprehensive training programs with prolonged post-workshop supervision and feedback on observed practice may help to sustain but not improve participants’ proficiency in MI. Potential explanations to the results and suggestions for future research are discussed.

## Introduction

Motivational interviewing (MI) is a client-centered, collaborative counseling style, which aims to facilitate clients’ motivation and commitment to behavior change by helping them to explore and resolve ambivalence [1]. MI is a style of communicating rather than a set of techniques. The underlying mind-set called the Spirit of MI is highly emphasized, and characterized by partnership, acceptance, compassion, and evocation (i.e., the belief that people have what is needed for change within them and that the therapist’s task is to help evoking it). Each of these concepts is manifested in practitioner behaviors towards the client [1].

A large body of research on MI has been conducted and meta-analyses generally present small to medium effect sizes across behavioral outcomes when compared to no-treatment or placebo conditions, with the strongest evidence in the treatment of addictive behaviors [2, 3]. However, there is a high degree of variability in effects across studies, even when the disorder and the patient population are the same, and there is a lack of evidence to fully explain this variability [1–3].

There has been an increasing demand for disseminating MI into clinical practice, which calls for attention to the practice and effectiveness of training in MI. Prior research shows significant differences in practitioners’ effectiveness in delivering MI [1]. Systematic reviews of MI training conclude that most studies report increases in participants’ MI skills post workshop [4–6]. However, studies display a high degree of heterogeneity in training format, participant characteristics and outcome measures, in addition to insufficient reporting of methodology, which makes it difficult to interpret the results [4–6]. A recent meta-analysis on MI skill retention after workshop concludes that studies without post-workshop training input reported eroding skills over a six-month period [7]. However, studies that included coaching or systematic feedback (i.e., feedback based on a structured measure of performance) after the workshop found sustained skills. According to the meta-analysis, combining systematic feedback and coaching was slightly more effective than coaching only. In addition, increasing the frequency and number of hours of post-workshop sessions as well as the time frame during which they occurred, had a positive effect on skill retention [7]. The authors highlight the need for more rigorous experimental designs and longer follow-up periods.

Potential factors influencing the outcome has been suggested in the literature, including practitioner baseline counseling skills, motivation to learn MI, empathy, educational level, lower endorsement of disease model beliefs and personality traits [4, 8–11]. Practitioner perceived self-efficacy (SE) might also have the potential to influence training outcome. SE refers to beliefs in one’s capability to successfully organize and execute specific courses of action [12]. It is a strong predictor of professional behavior, such as attaining goals, perseverance when facing obstacles and managing stress related to work [12, 13]. In a non-controlled study of the role of practitioner SE on implementation of an evidence-based parenting intervention in primary care, SE was positively associated with implementation in terms of proportion of target families receiving the full intervention [14]. Bohman and colleagues [15] studied SE among intervention and control nurses in the PRIMROSE childhood obesity prevention trial [16]. The intervention nurses who received MI training and training on dietary and physical activity (PA) interventions, demonstrated higher SE in terms of belief in ability to influence parents to promote healthy dietary and physical activity habits in their children.

The current study is an extension of a previous study on the level of proficiency after the MI training in the PRIMROSE trial [17]. When assessed after completed workshop and four sessions of supervision (two of which included systematic feedback), the nurses had not reached beginning proficiency thresholds on any of the indicators of MI proficiency and effects sizes were small. In the present study, the nurses had received five additional supervision sessions including systematic feedback. The overarching aim of the present study was to explore the potential benefits of an extensive MI post-workshop training package, delivered to nurses within the childhood obesity prevention trial PRIMROSE. We also wanted to investigate whether nurses’ initial level of empathy, years of experience as nurses in child health services (CHS), previous training in MI as well as efficacy beliefs before training would predict change in the level of MI proficiency.

## Method

### The PRIMROSE trial

The PRIMROSE trial was a population-based cluster-randomized intervention trial that started in 2008 and was completed in 2015 [16, 18]. The intervention aimed to prevent development of childhood obesity by promoting healthy eating and physical activity among children from infancy to their pre-school years (9 to 48 months of age), and their parents. Nurses in Swedish CHS delivered the intervention within the frame of MI, to parents attending these services. The intervention was manual-based and directed towards first-time parents. It was developed at Karolinska Institutet, Stockholm, Sweden by experts within the fields of psychology, childhood obesity, nutrition, and child health care. Parents were considered important role models for their children in developing healthy habits. Thus the PRIMROSE intervention focused on motivating parents to develop and/or maintain their own healthy eating and physical activity habits. At a later stage of the intervention and with increasing age of the children, there was a gradual shift of focus towards helping parents to promote healthy dietary and PA behaviors in their children using skills based on principles derived from learning theory and social cognitive theory (SCT). The intervention group was compared to a control group of parents receiving care as usual [16].

The trial took place at child health care centers (CHCs) in eight counties of Sweden. The CHCs are a well-established part of the nationwide Swedish child health promotion and health surveillance system. Almost all Swedish parents have regular contact with the services from the birth of their child up to 5.5 years of age. All parents and nurses eligible to be part of the trial were asked to provide written informed consent before enrollment. Approval of the PRIMROSE trial (2006/525-31/2) as well as the current study (2008/1256-32), was granted by the regional ethical review board in Stockholm, Sweden. The trial has been registered at the ISRCTN registry (ISRCTN16991919).

### Participants

All participating nurses were participants in the PRIMROSE trial, female and had specialist training in child and adolescent health nursing or district nursing. In Table 1, the intervention sample is described in more detail. At baseline, the trial nurses were compared to their colleagues working at the same CHCs in the eight counties, and the groups did not differ significantly on any of the variables [17]. Nurses in the control group were asked to record a session from routine practice corresponding to the timing of the first training session in the intervention group. However, due to recordings that were too short for reliable coding and recurrent topics that were not suitable for coding (associated with routine practice), the control group data could not be included in the analysis.

**Table 1:** Characteristics of the nurses after completed training and intervention sessions.

Characteristic	Post Workshop	After four supervised training sessions	After five supervised intervention sessions
*N*	51	39	33
Age in years, *M(SD)*	47.7(8.8)	47.5(8.8)	48.7(8.5)
Years in CHS, *M(SD)*	10.5(8.1)	11.15(8.2)	12.4(8.2)
Previous MI training (%)	54.9	59	57.6
Length of prior MI training in hours, *M(SD)*	22.9(37.7)	23.2(40.8)	24.7(44.9)

### MI training

The training addressed the learning stages 1 to 5 from the suggested eight stages in learning MI [19]: (1) the spirit of MI, (2) client-centered counseling skills, (3) recognizing and reinforcing change talk, (4) eliciting and strengthening change talk, and (5) rolling with resistance.

#### Workshop

Nurses received a 5-day workshop including an introduction to nutrition, PA, learning theory, and SCT, as well as training in MI. The MI training part of the workshop consisted of 3.5 days, with 8 hours of training per day. It was divided into two parts, 1.5 days + 2 days with an average interval of 17 days [17]. Seven workshops were conducted, each with an average of 10 participating nurses. The workshops were led by a senior clinical psychologist with extensive experience in leading MI workshops, and membership of the Motivational Interviewing Network of Trainers (MINT). Two more licensed clinical psychologists assisted as instructors. Didactic presentations and experiential exercises were used, in line with the training recommendations by the MINT, and covered the following topics: definition of MI; the evidence base; limitations of traditional advice-giving; MI principles; MI strategies; phase 1 and 2 of MI practice; learning MI; measurement of proficiency; predictors of client outcome; and theoretical considerations (for more details, see Bohman and colleagues [17]). The workshop also included role-play demonstrations by instructors, viewing of the Professional Learning Series video demonstrations [20], and handouts. Along with the lectures, the participants practiced the MI-skills in exercises and role-plays.

#### Supervision

The supervision was planned to last for 30 minutes and to be based on at least 20 minutes of an audio-recorded session. It was conducted by telephone by 10 MINT members with various professional backgrounds and an average of four years of experience as MI supervisors. Five of the supervisors were licensed clinical psychologists. Nurses received supervision on a total of nine sessions. The first four sessions were self-selected training sessions with parents of children of the ages 9–18 months and the last five sessions were conducted with parents of children enrolled in the PRIMROSE intervention group. The parents were randomly sampled among all intervention children at a specific CHC. The first training session and all the intervention sessions were coded according to the Motivational Interviewing Treatment Integrity (MITI) code [21] for use as systematic feedback, and the supervision was based on both the recording and the MITI protocol (which was sent to both nurses and supervisors before supervision). The supervisors were instructed to start the session by asking the nurses what area of MI skills they wanted to focus on. Feedback on the results from the MITI coding was embedded in the conversations that followed, combined with reinforcement of MI consistent behaviors and help to improve MI inconsistent behaviors.

The supervisors had access to telephone supervisory meetings once a month throughout the supervision period. From the start of the supervision in May 2008, five telephone supervisory meetings were held (60% attendance) that year, during 2009 nine meetings (50% attendance), during 2010 nine meetings (40% attendance) and during 2011, when the supervision period was ended, seven meetings were held (36% attendance). Notes from the telephone meetings were circulated to all the supervisors in order to create uniformity in interpretations of instructions and supervisory practices and provide solutions to up-coming problems.

#### Manual and website

The manual was based on learning theory, SCT, and MI. It contained session-by-session instructions and information. Parts of the manual contained information on healthy eating and PA that could be offered to the participating parent when needed. The manual also presented a short summary of the MI approach to help nurses maintain the skills they had learned at the workshop. The nurses received the manual by the end of the first part of the workshop and were able to ask questions about it during the second part, though the manual was not used as part of training. A website was developed to help additional learning and maintenance of achieved skills and knowledge. It contained the manual, lecture materials, video recordings of the workshop lectures and role-plays of MI principles and strategies when used by nurses in conversations with parents about healthy eating and healthy PA. The role-plays were delivered by professional actors and licensed clinical psychologists.

### Assessment

#### MI Proficiency

The MITI is a behavioral coding system that can be used both as a measure of clinician MI competence, and as a tool for feedback in clinical practice and training [21]. In this study, the Swedish version of the MITI, version 3.0 was used [22]. The MITI assesses global scores (the rater’s overall impression of the interviewer’s performance) of MI spirit, empathy, evocation, collaboration, autonomy/support and direction, using ratings on a 5-point Likert-type scale. MI-spirit is an aggregation of three other global scores (see Table 2 for details). MITI also includes frequency counts of specific behaviors (information giving, closed and open questions, simple and complex reflections, MI adherent behaviors, and MI non-adherent behaviors). The frequency counts can also be aggregated into summary scores (Table 2). Several scores were used as indicators of proficiency when evaluating the effect of the MI training in the current study; MI spirit, empathy, percent complex reflections, reflection-to-question ratio, percent MI adherent behaviors, and frequency of MI adherent and MI non-adherent behaviors.

**Table 2:** Formulas for calculation of MITI summary scores.

MITI summary score	Formula
MI Spirit	(Evocation + collaboration + autonomy/support)/3
Percent Complex Reflections	Complex reflections/ simple + complex reflections
Percent Open Questions	Open questions/open + closed questions
Reflection-to-Question Ratio	Simple + complex reflections/ open + closed questions
Percent MI Adherent behaviors	MI adherent behaviors/ MI adherent behaviors + MI non-adherent behaviors

Source: Moyers and colleagues [21]

MITI = Motivational Interviewing Treatment Integrity Code

The nurses made a note on the time point when the conversation started to focus on diet and PA, and from that point the sessions were coded for a maximum of 20 minutes. The raters were two persons proficient in MITI coding, with MITI training according to standards set by Moyers and colleagues [21], including 40 hours of training and bi-weekly participation in group-coding sessions. They were equally skilled and had practiced MITI rating for 4 and 5 years, respectively. Training and coding took place at the Motivational Interviewing Coding Laboratory (MIC Lab) at the Department of Clinical Neuroscience at Karolinska Institutet, Stockholm, Sweden. To ensure inter-rater reliability, 10–15% of all recordings sent to the lab were regularly double-coded.

Inter-rater reliability of the Swedish version of MITI 3.0 has been assessed with intra-class correlation coefficients (ICCs), using single measures and two-way mixed model with absolute agreement. ICCs ranged between .86 and 1.00 for global scores, which qualify as excellent [23]. Inter-rater reliability of the coding in the current study has been previously assessed based on the first 23 recordings of the first PRIMROSE trial session [17]. ICCs across global scores and behavior counts ranged from .35 to .85. Percent complex reflections qualified as poor (.35), empathy and MI adherent behaviors as fair (.55 and .47, respectively), while remaining five (63%) ICCs were in the adequate (MI spirit, empathy, MI non-adherent behaviors) to excellent (percent open questions, reflection-to-question ratio, MI adherent behaviors) range.

An analysis of the sensitivity of the MITI in identifying practitioner behavior change between baseline and after the workshop showed significantly higher scores on a majority of the indicators [24]. These results were replicated in an analysis of the Swedish version of MITI [23]. When assessing MI competence, thresholds for indicators of proficiency are commonly used. However, these thresholds are based on expert opinion and at present there exists no validity data to support them [21].

#### Self-efficacy

Nurses’ efficacy beliefs were assessed using an instrument developed for the PRIMROSE trial [15]. It is intended to address nurses’ efficacy beliefs in influencing parents to promote healthy dietary and physical behaviors in their children. The questionnaire is comprised of 18 questions with responses on an 11-point Likert-type scale, from 0 to 10.

### Procedure

**Fig 1:**
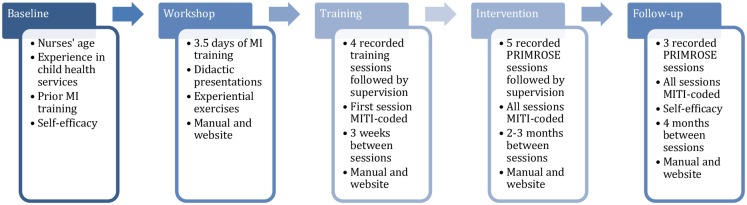
Procedure overview. Training session = self-selected; trial session = randomly selected PRIMROSE session. The first training session was planned to take place 3 weeks after the workshop and the first follow-up session 4 months after the last intervention session.

The participating nurses answered a questionnaire about previous work experience within the CHS, and former education and training. They were then randomized to either the intervention or control group. Nurses not participating in the PRIMROSE trial at the CHCs in the participating counties were also asked to fill in the questionnaire in order to check for any potential differences. After filling in the questionnaire, nurses in the intervention group enrolled for MI training. The training sessions and the following five intervention sessions were audio-recorded. The first training session and all the intervention sessions were then coded using the MITI. After completion of the MI training and all sessions of supervision, nurses were asked to record three additional follow-up sessions that were MITI-coded in order to assess retention of skill levels. For details on intended time between different recordings of sessions, see Fig 1.

### Data analysis

Data were screened for normality and analyzed using the Statistical Package for the Social Sciences (SPSS) (Version 22, SPSS Inc., Chicago, IL.). Group comparisons were made using independent *t*-tests and within-subject changes over time were analyzed using paired *t*-tests. Cohen’s *d* was used for calculation of effect sizes. Multiple regression analyses were conducted to investigate potential predictors of improvement in MI proficiency between four and nine sessions of supervision.

## Results

### Dropout analysis

The number of participating nurses in the present study decreased in the later stages of data collection due to dropout. After the workshop, 51 nurses recorded and submitted a session for MITI coding. At the next assessment point (i.e., after four sessions of supervision) 39 nurses (76%) provided recorded samples. A total of 33 nurses (65%) completed the training and all nine sessions of supervision as well as provided recordings of a 10th session. When comparing the dropout group with the remaining participants after four sessions of supervision no significant differences were found regarding age, years in CHS, length of prior MI training in hours, or MITI-scores after workshop (for details on participant characteristics, see Table 1). When comparing the two groups after nine sessions of supervision, there were still no significant differences except for number of years within the CHS. On this variable the nurses remaining in the trial had significantly longer work experience than the non-completers, *t(49)* = 2.8, *p* = .019, Cohen’s *d* = 0.83.

### Timing of trial and supervision sessions

Mean length of time between the sessions from session 5 to 10 was 4.6 months (*SD* = 1.4, range = 3.0–9.4). The total mean time between session 5 and 10 was 23.2 months (*SD* = 7.2, range 15.2–46.8), instead of the intended 15 months. Mean time between the ninth supervision session and the last follow-up session was 14.9 months (*SD* = 5.3, range = 7.8–34.8) instead of the intended 12 months. Regarding length of recorded sessions the nurses managed fairly well to follow the instructions; mean session length was 19.61 minutes (SD = 0.70, range = 16.84–20.00).

### Proficiency in MI after completion of supervision and systematic feedback

After receiving the additional five sessions of supervision and systematic feedback and thereby completing the whole MI training package, the nurses remained on approximately the same levels of proficiency as measured after four training sessions of supervision, mean differences ranging from -1.12 to 0.10 across indicators of proficiency (see Table 3). Paired *t*-tests showed non-significant differences on six of the eight MITI indicators with *t*-values ranging from *t*(32) -1.08 to 0.73, and *p*-values ranging from 0.29 to 1.0. The difference on two of the indicators were significant; percent complex reflections (*t*(32) = 3.64, *p* = .001, *d* = 0.67), suggesting an improvement, and MI -adherent behaviors (*t*(32) = -2.82, *p* = .008, *d* = -0.49), suggesting a deterioration across time (for more information, see Table 3).

**Table 3:** Proficiency in motivational interviewing in nurses (*n* = 33) after four and nine sessions of supervision.

MITI indicator of proficiency	Mean (*SD*) after four sessions of supervision	Mean (*SD*) after nine sessions of supervision	Mean difference (*SD*)	*t*	*p*	Cohen’s *d* effect size
MI spirit	2.79 (0.60)	2.87 (0.58)	0.08 (0.65)	0.73	.474	0.13
Empathy	2.79 (0.60)	2.79 (0.78)	0.00 (0.83)	0.00	1.00	0.00
Percent complex reflections	0.14 (0.13)	0.25 (0.15)	0.11 (0.16)	3.64	.001	0.67
Percent open questions	0.39 (0.19)	0.37 (0.18)	-0.02 (0.27)	-0.49	.625	-0.08
Reflection-to-question ratio	0.83 (0.63)	0.85 (0.48)	0.02 (0.67)	0.175	.862	0.03
Percent MI adherent behaviors	0.69 (0.40)	0.57 (0.50)	-0.13 (0.67)	-1.08	.288	-0.19
MI adherent behaviors	2.36 (1.82)	1.24 (1.46)	-1.12 (2.29)	-2.82	.008	-0.49
MI non-adherent behaviors	0.48 (0.83)	0.45 (1.03)	-0.03 (1.38)	-0.13	.900	-0.02

MITI = The Motivational Interviewing Treatment Integrity Code

The results were also analyzed in terms of proportion of participants reaching beginning proficiency thresholds on the different MITI indicators. Only one participant of 33 reached beginning proficiency thresholds on all variables. The percentage of nurses reaching the proficiency level ranged from 18.2 to 54.5% across indicators, with the highest percentage for percent open questions, reflection-to-question ratio and percent MI adherent behaviors (see Table 4).

**Table 4:** Nurses reaching beginning proficiency thresholds (*n* = 33).

MITI indicator of proficiency	Beginning proficiency thresholds	% of nurses proficient after nine sessions of supervision	95% confidence interval
Global ratings (MI spirit + Empathy/2)	3.5	21	6–36
Percent complex reflections	0.4	18	4–32
Percent open questions	0.5	24	9–40
Reflection-to-question ratio	1.0	27	11–43
Percent MI adherent behaviors	0.9	55	37–72

MITI = Motivational Interviewing Treatment Integrity Code.

Using independent *t*-tests, nurses reaching beginning proficiency (n = 6–18) on the different indicators where compared to those that did not (n = 15–27) in terms of prior work experience and MI training, initial level of empathy as measured after the workshop and self-efficacy at baseline. No significant differences were found.

### Predictors of change in proficiency

Multiple regression analyses were conducted to find potential predictors of change on MITI indicators between the assessments after four and after nine sessions of supervision. Independent variables were SE at baseline, level of empathy as measured after the workshop, previous MI-training, number of years within CHS and time elapsed between session 5 and 10 when nurses had received all supervision. None of these variables significantly predicted change on any of the MITI indicators.

### MI proficiency at follow-up

Paired t-tests were conducted comparing proficiency levels after nine sessions of supervision and at the last follow-up session. On seven out of eight indicators there were no significant differences, however, MI-spirit had a mean of 3.13 (SD = 0.46) and had increased significantly, *t*(27) = 2.52, *p* = .018, *d* = 0.49.

## Discussion

The current study explored the levels of MI proficiency after the nurses in the PRIMROSE trial had received the complete MI training package including the workshop and nine sessions of supervision, of which six included systematic feedback. The prolonged supervision and systematic feedback did not increase MI proficiency and the results were not predicted by nurses’ efficacy beliefs at baseline, level of empathy directly after the workshop, years of experience within CHS, previous training in MI, or time elapsed between session 5 and 10. Proficiency remained on the same level on six out of eight MITI indicators, percent complex reflections had increased significantly while the number of MI adherent behaviors had significantly decreased (both with effects of medium size). At follow-up, 14 months after completion of the supervision, MI spirit had increased significantly showing an effect approaching medium size level, but there were no significant differences on the other seven indicators. The results were similar to the results of the study by Bohman and colleagues [17] on MI proficiency among the nurses after four sessions of supervision.

Only one nurse reached beginning proficiency thresholds on all MITI indicators and the percentage reaching the thresholds on the different indicators ranged between 18% and 54.5%. There were no significant differences between the nurses that had reached specific thresholds and nurses that had not, in terms of prior work experience and MI training, initial level of empathy or self-efficacy.

We have chosen to use the term “supervision” when referring to the post-workshop training received by the nurses in the current study. However, there is not a consensus within the field on what term to use, and sometimes the term “coaching” is used instead. By “supervision” in the current study we refer to a conversation between the supervisor and the nurse, where the supervisor provide feedback on an MITI coded session, reinforce MI consistent behaviors and help to improve MI inconsistent behaviors.

Previous research shows that in most cases a workshop leads to significant improvement of MI proficiency [4–6]. If we suppose that the nurses in the current trial did improve their MI proficiency as a result of the workshop, however not to a high level, the proficiency measured at later time-points shows that this achieved level of proficiency was sustained both after four sessions of supervision [17] and after nine sessions. A meta-analysis on sustaining MI skills after workshop reported eroding skills in studies where post-workshop coaching/supervision and/or systematic feedback were not included [7]. In the current trial the proficiency levels were sustained but did not improve. Maybe the supervision did help skill retention to some extent but the lack of pre-assessment and control group only allow for speculation. The current trial had an explorative focus, aiming to understand more about the potential benefits of training nurses to use MI as a tool for preventing childhood obesity, but a more large-scale study would preferably also include a pre-assessment.

To our knowledge, few studies have experimentally examined what post-training efforts are most efficient. In a study comparing different MI training methods [9] additional coaching and/or feedback after the workshop showed improved proficiency. Outcome in terms of MI proficiency did not differ significantly between the three different supervisory settings, and the combination of both coaching and systematic feedback didn’t produce better scores than either of the methods alone. A study comparing tape-recorded supervision and tele-conferences found that the outcome of the different supervisory conditions did not differ significantly [25]. In the meta-analysis by Schwalbe and colleagues [7], exploring what level of post-training input is needed to sustain MI skills, the authors concluded that three to four contacts, comprising of at least five hours in total over a 6-month period are sufficient for skill retention.

The MITI proficiency thresholds were used to evaluate MI skill levels of the participants, although these thresholds are based on expert opinion and not validity data [21]. Only two studies have shown results with participants reaching beginning proficiency on all indicators [8, 9]. Nonetheless, the proportion reaching beginning proficiency levels in the current trial is lower compared to previous trials with equivalent training packages and objectively coded sessions with clinical samples [8, 9, 25]. This is unexpected also considering that the nurses in the current trial participated voluntarily, and a majority of them had previous MI training.

The results of this study should be interpreted with caution. The sample size is small, thus providing low power to detect any small differences. In addition, the homogeneity of the sample makes it difficult to find potential predictors of change in MI proficiency. The lack of pre-assessment and control-group does not allow us to draw conclusions on causality.

When interpreting the results it is also important to consider that the inter-rater reliability for percent complex reflections, the only indicator that had significantly improved, was estimated as poor according to the ICC scores. However, MI spirit, the indicator that had improved significantly at follow-up, had adequate reliability.

In the preceding study by Bohman and colleagues [17], the low level of proficiency among the nurses after workshop and four sessions of supervision and systematic feedback were discussed in detail. Several possible explanations were mentioned, for example digressions from instructions on timing between sessions, the manual-based character of the PRIMROSE trial and the demand on nurses to provide mandatory information according to the standards of CHS (in contrast to the client-centered MI-approach), stress due to workload, and possible lack of organizational support. Furthermore, it was reported from nurses that parents often brought their child with them to the meeting, which sometimes made it difficult for the nurses to keep focus during the conversation. Nurses also reported problems connected to the preventive nature of the intervention and already motivated parents with established healthy behaviors, making it difficult to detect target behaviors for MI interventions. The fact that the nurses didn’t improve after the additional five supervision sessions could be explained by the factors mentioned above [17].

This trial has both limitations and strengths and so far mainly the limitations have been mentioned. However, the trial also has considerable strengths. The participants were offered a comprehensive training package, including nine sessions of post-workshop supervision with six of them including systematic feedback. Highly experienced and skilled supervisors performed the supervision. The nurses’ skill levels were objectively measured using the MITI coding system at nine time-points including long-term follow-up. The nurses participated voluntarily, and a majority of them had some former MI training and could be expected to be receptive to training. In addition, although the clinical setting of the trial led to some challenges, as mentioned above, trials embedded in routine health and medical care services may provide important new knowledge about factors influencing implementation, both on an organizational and individual level.

More research on different MI-training components and their significance for achieving and maintaining proficiency is needed. There is a lack of knowledge about what specific elements of post-training supervision/feedback are the most effective for both skill retention but ultimately also skill improvement. We also need to further investigate whether there are subgroups of people that are more susceptible to training, both in terms of personality traits and cognitive ability as well as factors such as prior work experience, educational level and organizational support. The nature of the prior education may also have impact; maybe it is more difficult to learn MI for professionals with educational backgrounds focusing on giving advice and taking the role of an expert, an approach very different to the spirit of MI. There is also a need for more research on the application of MI to preventive interventions, when a target behavior might be more difficult to find, and the clients might already be fairly motivated. Could MI be used to target the maintenance of a desired behavior and for prevention of future relapse? The results from the current study suggest that MI training in a context of prevention may present additional challenges, both in delivering MI but also regarding the assessment of MI competence.

### Conclusion

In a non-controlled longitudinal study of nurses in CHS who received extensive post-workshop supervision in MI, there was no decline in proficiency after workshop and potentially an increase in one area at follow-up. However, the general level of proficiency was low, and even if there was a tendency towards sustained skills, the overall conclusion is that a comprehensive MI training package might not be enough to provide sufficient MI proficiency for nurses within the context of childhood obesity prevention. However, the result could potentially be explained by contextual factors, which impeded the nurses to practice MI. Further research is needed, specifically examining practice-based MI training within in the field of primary care and health promotion.

## Supporting Information

S1 File S1_File.xlsx. (XLSX)

## Abbreviations

MI: motivational interviewing
MITI: motivational interviewing treatment integrity code
SE: self-efficacy
PA: physical activity
CHS: child health services
SCT: social cognitive theory
CHC: child health care center
MINT: motivational interviewing network of trainers
MIC Lab: motivational interviewing coding laboratory
